# Biodegradable ether amines for reverse cationic flotation separation of ultrafine quartz from magnetite

**DOI:** 10.1038/s41598-023-47807-0

**Published:** 2023-11-23

**Authors:** José Tadeu Gouvêa Junior, Vitalis Chipakwe, Laurindo de Salles Leal Filho, Saeed Chehreh Chelgani

**Affiliations:** 1 Department of Mining and Petroleum Engineering, Polytechnic School, University of São Paulo, Avenida Professor Melo Moraes, 2373, Cidade Universitária, São Paulo, SP 05508-900 Brazil; 2 Minerals and Metallurgical Engineering, Swedish School of Mines, Department of Civil, Environmental and Natural Resources Engineering, Luleå University of Technology, 971 87 Luleå, Sweden

**Keywords:** Engineering, Chemical engineering

## Abstract

A considerable amount of ultrafine magnetite as the iron source will end up in the tailing dams since the magnetic separation process markedly drops as the particle size. Cationic reverse flotation could be one of the main alternatives for recovering ultrafine magnetite. As a systematic approach, this study explored the flotation efficiency and interaction mechanisms of two biodegradable ether amines (diamine and monoamine) to separate ultrafine quartz from magnetite (− 20 µm). Several assessments (single and mixed mineral flotation, zeta potential, contact angle, surface tension measurement, turbidity, and Fourier transform infrared) were conducted to explore the efficiency of the process and the interaction mechanisms. Results indicated that ether diamine and monoamine could highly float ultrafine quartz particles (95.9 and 97.7%, respectively) and efficiently separate them from ultrafine magnetite particles. Turbidity assessments highlighted that these cationic collectors could aggregate magnetite particles (potentially hydrophobic coagulation) and enhance their depression. Surface analyses revealed that the collector mainly adsorbed on the quartz particles, while it was essentially a weak interaction on magnetite.

## Introduction

The extraordinary growth in steel demand has emerged in iron mines for further production. Magnetite and hematite (iron oxides) are the primary iron resources mainly upgraded by magnetic separators before they are subjected to steelmaking processes. Nevertheless, magnetic separators are limited in processing fine and ultrafine particles. Ultrafine particles may not be effectively captured by magnetic separators due to their small size or being covered with gangue phases. Therefore, a large amount (5–20% of the valuable minerals in the feed) of fine and ultrafine magnetite and hematite particles will end up in the tailing dams^1–4^.

Flotation separation (direct or reverse) is the most common method for processing iron oxides in a typical size range of − 150 + 40 µm. However, the flotation of ultrafine particles (− 40 µm) is limited since these particles show a low level of collision with air bubbles in flotation systems, jeopardizing further adhesion to bubbles and eventual flotation^5,6^. Few investigations have addressed the flotation beneficiation of ultrafine hematite particles and presented the process separation efficiency. The studies carried out considered different flotation reagents (collectors, depressants, etc.) and used them to improve the recovery process (Table 1).

**Table 1:** Ultrafine hematite flotation processes.

Minerals	Particle size (µm)	Reagents	Process	Recovery (%)	References
Hematite (pure)	d_50:_ 4	Sodium oleate (collector)	Direct flotation	90	^7^
Hematite (pure)	d_50_: 19	Sodium oleate (collector)	Direct flotation	81	^8^
Hematite-quartz	− 25	Dodecylamine (collector)	Reverse flotation	80	^9^
Hematite-quartz	− 20	Waxy corn starch and corn dextrin (depressants) Dodecylamine (collector)	Reverse flotation	82	^10^

However, the upgrading of ultrafine magnetite flotation was rarely addressed. Chipakwe et al. examined the effect of a polyacrylic acid-based grinding additive (GA) through flotation separation of magnetite from quartz (size fraction of − 106 + 38 µm) using an ether amine-based collector and indicated that using GA can reduce collector consumption^11^. Jintian et al. indicated that the reverse flotation-hydrogen reduction could be used to produce the ultra-pure magnetite concentrate (72.12% Fe and 0.19% SiO_2_) when d_80_ was − 37 μm^12^. It was documented that the electrolysis process would enhance the flotation separation of quartz from magnetite (d_50_: 22.5) when sodium oleate was used as a collector^13^. Generally, typical flotation reagents have been reported to be not effective in ultrafine particle flotation due to their high surface area, leading to challenges in achieving optimal flotation conditions and reagent dosages for their beneficiation^11–13^.

Cationic surfactants such as ether diamine (Lilaflot®811M) and ether monoamine (Lilflot®919) are collectors that have shown high efficiency in processing ultrafine particles^14–16^. Li et al. (2016) indicated the possibility of reverse flotation separation of silicate from coal particles by using Lilaflot®. Lilaflot® could successfully float fine quartz (− 56 μm) and reduce the coal ash content by 35%^15^. They also concluded that coal ultrafine particles (− 45 μm) could adsorb more Lilaflot® due to their larger specific surface area, leading to less collector left in the pulp to float silica particles^16^. Although they are environmentally benign and eco-friendly (compared to other amines), surprisingly, there is no evidence of their application in the flotation separation of ultrafine silicates from iron oxides in the literature. From this concise literature review, using biodegradable ether amines presents an opportunity to improve the rejection of ultrafine silicates from iron oxides via reverse flotation. Thus, this study explores the potential use of ether amines by exploring two different reagents in the Lilaflot® range (ether diamine and ether monoamine produced by Nouryon), which are candidates for reverse cationic flotation to separate quartz from magnetite. Various flotation conditions (single mineral and mixed mineral batch test with mixed minerals) and factors (separation efficiency and flotation kinetics) were conducted to explore the collection effectiveness of the ether amines. Analytical methods, including zeta potential, turbidity, wettability, and FT-IR spectroscopy measurements, were performed to understand better the interaction observed in flotation separation. The reported findings could help develop novel approaches to address the longstanding challenge in ultrafine particle flotation.

## Materials and methods

### Materials

**Figure 1:**
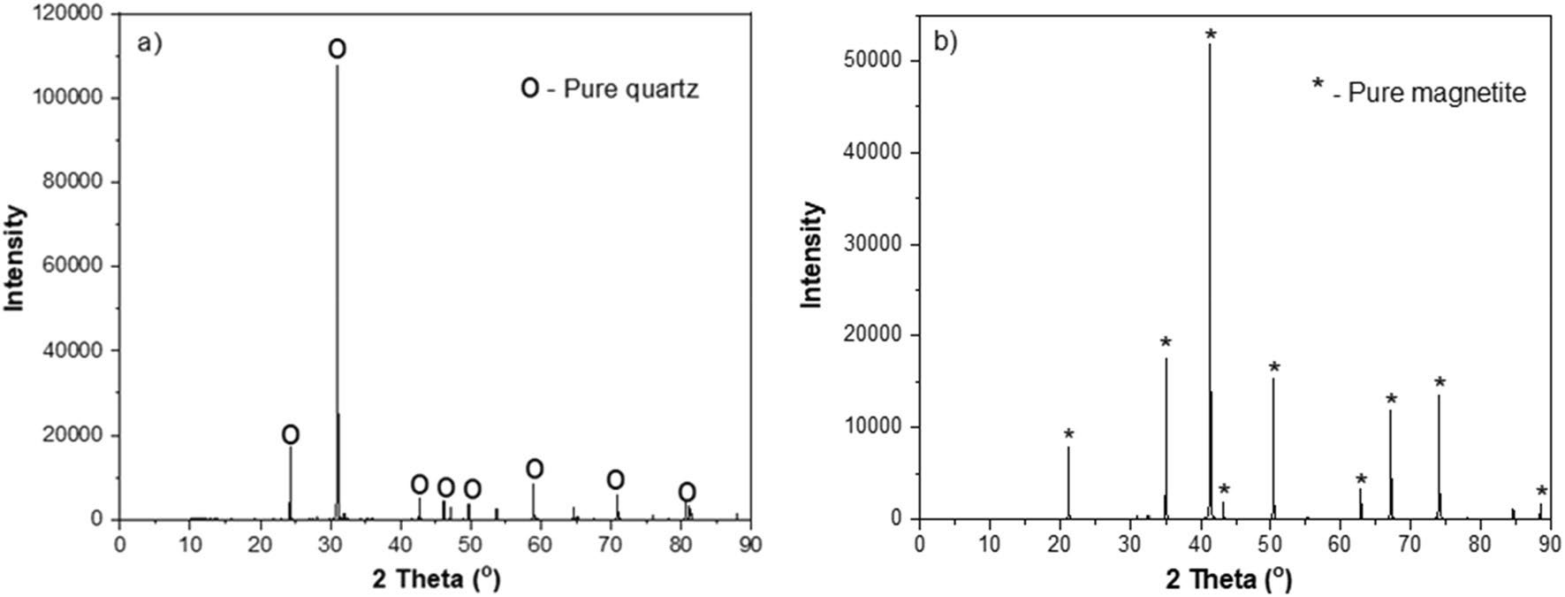
XRD pattern for the magnetite sample.

Pure quartz (> 99% SiO_2_) and magnetite (> 96% Fe_3_O_4_) samples (Fig. 1) were obtained from VWR, Sweden. The samples were ground using a laboratory-scale ball mill to produce flotation feed. The flotation feed had a d_80_ = 16.7 µm, a d_50_ = 7.5 µm, and a mean diameter of 9.55 µm. Nouryon (Sweden) provided two different ether amines, Lilafot® 811 M and Lilaflot® 919. The degradation of Lilaflot® 811 M and 919 is 10 and 41% in 28 days, respectively. Furthermore, corn starch (depressant) and pH modifiers were used in the flotation tests. Deionized water was used in all experiments unless otherwise stated.

### Flotation tests

#### Single-mineral-flotation

Single-mineral flotation for pure quartz was performed using a mini flotation cell (Clausthal cell). In each flotation experiment, 7.5 g of the mineral sample (− 20 µm) was suspended in deionised water to complete an overall volume of 150 cm^3^. The predetermined amount of reagents (depressant and collector) was added to the slurry and conditioned for 10 min (for depressant) plus 5 min for the collector after adjusting the pH of the suspension. Caustic starch was used as a depressant to inhibit magnetite floatability. For each set of experiments, a fresh 1% solution of alkaline starch was prepared at 1:4, starch: NaOH ratio. The pH was adjusted by adding 1.0 M NaOH or 1.0 M HCl. The flotation was conducted for 5 min, scraping every 10 s. The floated and sunken products were collected, dried, and weighed, and the recovery was calculated based on the dry weight. Each experiment was carried out in duplicate, and the average was reported. Because the experimental results fit a first-order kinetic model, the flotation rate constant *k* plus the maximum attainable recovery ${R}_{\infty }$ were determined from Eq. (1) through curve fitting for results obtained with both collectors.

1 $${R}_{(t)}= {R}_{\infty } (1-{e}^{-kt})$$

where R_(t)_ = cumulative recovery of quartz in the froth in a time t (%); ${R}_{\infty }$ = maximum cumulative recovery of quartz as time tends to infinity (%); k = flotation rate constant (s^−1^).

#### Mixed mineral flotation

Mixed mineral flotation of the model ore was carried out using a Clausthal cell. The model ore consisted of 5.0 g magnetite and 2.5 g quartz (ratio 2:1). For each test, 7.5 g of the mixture was used together with deionized water. The collector concentration was varied from 50 to 100 mg/L (translates to 50–500 g/t) whilst the depressant was fixed at 100 mg/L, which translates to 1000 g/t. Conditioning was carried out for 10 min, followed by a 5-min flotation test. The floated and sunken products were collected, dried, and weighed, and the recovery was calculated based on the dry weight. Each experiment was carried out in duplicate, and the average was reported. Each test's separation efficiency (SE) was calculated using Eq. (2)^17,18^.

2 $$S.E \left(\%\right)= \frac{c(f-t)(c-f)(100-t)}{f{\left(c-t\right)}^{2}(100-f)}\times 100$$

where *f*, *c,* and *t* are the feed, concentrate, and tail grades of iron, respectively, a higher S.E value indicates a better separation efficiency of the process.

### Zeta potential measurements

Zeta Potential measurements were performed using a Malvern zeta nano sizer (nano-ZS90). A mass of 50 ± 1 mg of quartz or magnetite (< 20 µm) was added to a 100 ml collector solution with the desired pH (adjusted by adding NaOH or HCl) and collector concentrations (different for each experiment). The suspension was conditioned by stirring for 5 min, and the desired pH was kept constant. Because magnetite bears Fe^2+^, the E_h_ of the suspension was measured to assess the degree of surface oxidation indirectly. All experiments were carried out at 22 ± 1 °C with a background electrolyte of 10^–3^ M NaCl in distilled water, and the results were the arithmetic mean of measurements made in triplicate. The zeta potential of quartz and magnetite particles was measured at pH 9,0 by varying the concentration (2, 5, 10, 20, 30, 50 mg/L) of the collectors (ether diamine or ether monoamine). Complementary measurements were performed at a collector concentration of 30 mg/L and by varying the pH of the solution (2, 4, 6, 8, 10, 11).

### Turbidity of mineral suspensions

The turbidity of suspensions provides information on the state of dispersion or aggregation of powders in a liquid. Knowing these two phenomena, one can assess the effectiveness of the interaction between minerals and reagents in an aqueous solution. Turbidity measurements were performed with a Thermo Scientific turbidity meter (Model: AQUAfast II Orion AQ2010). A mass of 1.0 g of each pure mineral (< 20 µm) was added to a 100 mL solution, adjusted to 9.0, and then stirred for 3 min. After that, the suspensions were kept at rest for 5 min to allow particle settling. Then 10 ml of supernatant (at the same height for all solutions) was picked and submitted to turbidity measurements. All measurements were carried out in triplicate at 22 ± 1 °C.

### Wettability 

The wettability of quartz and magnetite was characterized by contact angle measurements in the presence of reagents. Goniometer DSA25 (supplied by Kruss, Germany) was adopted to carry out contact angle measurements using the CBM (Captive Bubble Method) after a conditioning time of 5 min. To measure the contact angle, an air bubble was gently placed on a quartz (or magnetite) plate using a glass syringe endowed with a needle of diameter 0.487 mm. The surface of the mineral plate was preconditioned with a collector solution of known concentration. The measurements were conducted in triplicate at 22 ± 1 °C. To achieve an accurate contact angle measurement, the intersection points (the system's three-phase contact points between the fitted bubble shape and the baseline) are automatically identified by the equipment software. The Young–Laplace method was adopted to fit the bubble boundary line/contour prior to any angle measurement. After a time interval of 5 min, the equilibrium value of the contact angle (θ_eq) was registered. Results on θ_eq for quartz and magnetite contact were obtained at different concentrations of typical collectors (2, 5, 10, 20, 30, 50 mg/L) at pH 9.0. All measurements were carried out in an indifferent electrolyte solution (NaCl 10^−3^ M) in distilled water.

According to Eq. (3), the three-phase equilibrium between the air bubble, the mineral surface and the aqueous medium can be described by the contact angle ($\theta$) and the respective interfacial tensions: liquid–gas (${\gamma }_{LG})$, solid–gas (${\gamma }_{SG}$) and solid–liquid (${\gamma }_{SL}$). The displacement of the adsorbed surface water by an air bubble is controlled by the change in free energy per unit area corresponding to the particle/bubble attachment process (${\Delta G}_{attachment}$), as depicted by Eq. (4). This free energy change can be expressed in terms of the contact angle—Eq. (5), and the attachment process is seen to be spontaneous for all contact angles greater than zero^19,20^. According to Fuerstenau and co-workers^20^, the free energy change related to particle/bubble attachment (${\Delta G}_{attachment}$) can also be described in terms of the work of adhesion of water to the mineral surface (W_a_) versus the work of cohesion (Wc) of water itself, according to Eq. (6). W_a_ is defined as the work required to remove water from the solid surface, leaving an adsorbed water layer in equilibrium with a saturated gas phase, while W_c_ is the energy required to create a new air/water interface (144 mJ/m^2^ at 25 °C). As shown in Table 2, W_a_ and W_c_ are the sum of the polar plus dispersive (nonpolar) contributions. Because particle/bubble attachment depends on the inequality Wa < Wc, one can conclude that bare quartz particles cannot attach to bubbles and float. Therefore, collectors such as alkyl ether amines are needed to make particle/bubble attachment feasible prior to further levitation.

**Table 2:** Energy components for Wc and Wa for the quartz/water system^20^.

Components		W_c_* (mJ/m^2^)	Wa** (mJ/m^2^)
Polar	Ionization energy due to Coulombic attractive forces at the quartz/water interface	–	368
	The hydrogen bond energy is due to the dipole interaction between water/water molecules or water/quartz surface	102	
Nonpolar	Dispersion energy due to induced dipole interactions between water/water molecules or water/solid surface	44	102
Total		146	470

*Between water molecules.

**Between water and the surface of the quartz.

3 $${\gamma }_{SG}= {\gamma }_{SL}+ {\gamma }_{L/G}\,\,\mathrm{cos}\,\,\theta $$

4 $${\Delta G}_{attachment}= {\gamma }_{SG}-({\gamma }_{SL}+{\gamma }_{LG})$$

5 $${\Delta G}_{attachment}= {\gamma }_{LG} \left(\mathrm{cos}\,\,\theta -1\right)$$

6 $${{\Delta G}_{attachment}=W}_{a}-{W}_{c}$$

The arithmetic mean of the measured contact angle values and their standard deviations for any experimental condition was applied in Eqs. (7) and (8), to calculate the adhesion work (W_a_) of the aqueous medium to the surface of the minerals and the free energy of particle/bubble attachment (${\Delta G}_{Attachment}$). The surface tension of the different collector solutions versus the concentration was measured using a K100 force tensiometer (Kruss, Germany). The standard deviation of W_a_ (${\sigma }_{{W}_{a}}$) and ${\Delta G}_{Attachment}$ (${\sigma }_{{\Delta G}_{attachment}}$) were calculated by using Eqs. (9) and (10), respectively.

7 $${W}_{a}={\gamma }_{LV}(\mathrm{cos}\,\,\theta +1)$$

8 $${\Delta G}_{attachment}={\gamma }_{LV}(\mathrm{cos}\,\,\theta -1)$$

9 $${\sigma }_{{W}_{a}}=\sqrt{{\sigma }_{\gamma }^{2}\left(1+\mathrm{cos}\,\,\theta \right)^{2}+{\gamma }^{2}{\sigma }_{\theta }^{2}{\mathrm{sin}}^{2}\,\,\theta }$$

10 $${\sigma }_{{\Delta G}_{attachment}}=\sqrt{{\sigma }_{\gamma }^{2}\left(\mathrm{cos}\,\,\theta -1\right)^{2}+{\gamma }^{2}{\sigma }_{\theta }^{2}\mathrm{sin}^{2}\,\,\theta }$$

where W_a_ = work of adhesion [mJ/m^2^], ${\Delta G}_{attachment}$ = free energy of particle/bubble attachment [mJ/m^2^], θ = contact angle [degree, dimensionless], γ_LV_ = surface tension of the different solutions [mN/m], σ_θ_ = contact angle standard deviation [radian, dimensionless], and σ_γ_ = surface tension standard deviation [mN/m].

### FT-IR spectroscopy measurements

Diffuse reflectance (DR) and attenuated total reflectance (ATR) FTIR spectroscopy were used to analyse the surface of the samples. An IFS 66 V/S instrument and a Vertex 80v instrument were considered, respectively (Bruker Optics, Ettlingen, Germany), under vacuum conditions (below 7 mbar). For the analysis, each mineral was ground to approximately − 2 µm by an agate mortar and pestle. A 2.0 g aliquot was treated with predetermined flotation reagents and conditioned for 40 min at pH 10. The solid samples were thoroughly washed with deionized water. After washing and vacuum drying at 35 °C for 24 h. The dry sample (ca. 10 mg) was mixed with infrared spectroscopy grade potassium bromide (KBr, Merck/Sigma-Aldrich, ca. 390 mg) and manually ground using an agate mortar and pestle until a homogeneous mixture was achieved. Spectra were recorded in the 400–4000 cm^−1^ range at 4 cm^−1^ spectral resolution, and 128 scans were co-added, using pure KBr as the background under the same parameters^11^.

## Results and discussion

### Flotation tests

#### Single-mineral-flotation

**Figure 2:**
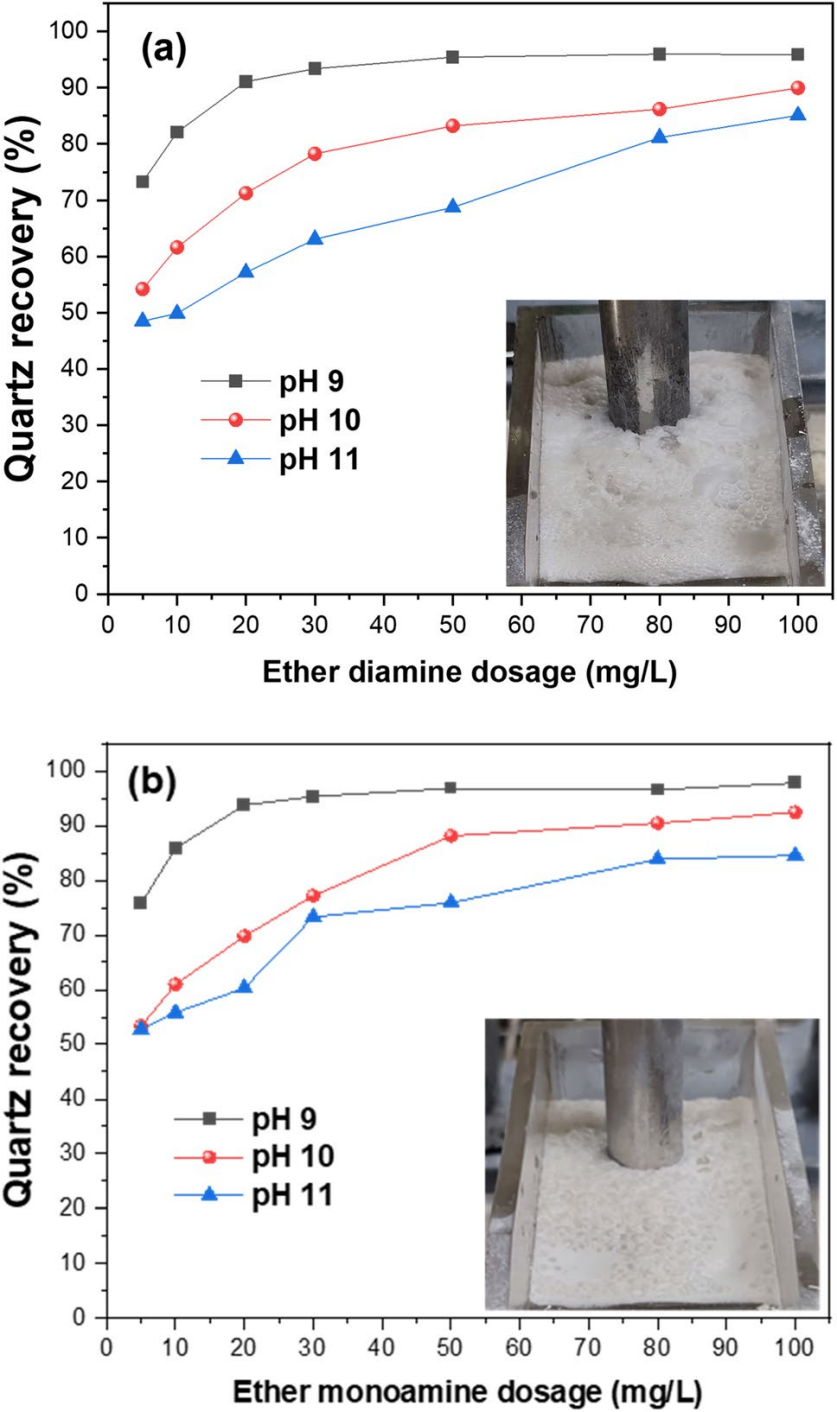
Single mineral flotation of quartz as a function of collector concentration of (**a**) ether diamine and (**b**) ether monoamine at varying pH.

The results obtained from the flotation experiments conducted with quartz indicated (Fig. 2) that the collector dosage or the pH of the solution affects the flotation recovery. Superior recoveries were evident at pH 9 compared to pH 10 and 11, regardless of collector type and dosage. For all cases, quartz increased with increasing ether diamine concentration to a maximum of 95.9, 90.0, and 85.1% at pH 9, 10, and 11, respectively. In the case of ether monoamine (Fig. 2b), relatively higher recoveries were recorded with a maximum of 97.7, 92.4, and 86.5% at pH 9, 10, and 11, respectively. At pH 9.0, the flotation response is enhanced, which could be explained by the condition that favors collector adsorption through coulombic attraction^21^. Furthermore, the observed decrease in quartz recovery as the flotation pH increased from pH 9 to pH 10 could be attributed to the reduced degree of dissociation (< 50%) of ether amines as the pH increases and the ionized species deplete relative to the molecular species^19,21^. At the best pH 9, the quartz recovery plateaued at a collector concentration of 50 mg/L, with superior flotation reported for ether monamine compared to diamine. Visual inspection of the froth properties yielded by both collectors is evident in Fig. 2. In Fig. 2a, the ether diamine froth had polyhedral-shaped bubbles, which appeared to be dry. The ether monamine froth showed more spherical and voluminous bubbles surrounded by thick liquid boundaries, indicating wet froth^19,22^.

#### Flotation kinetics

**Figure 3:**
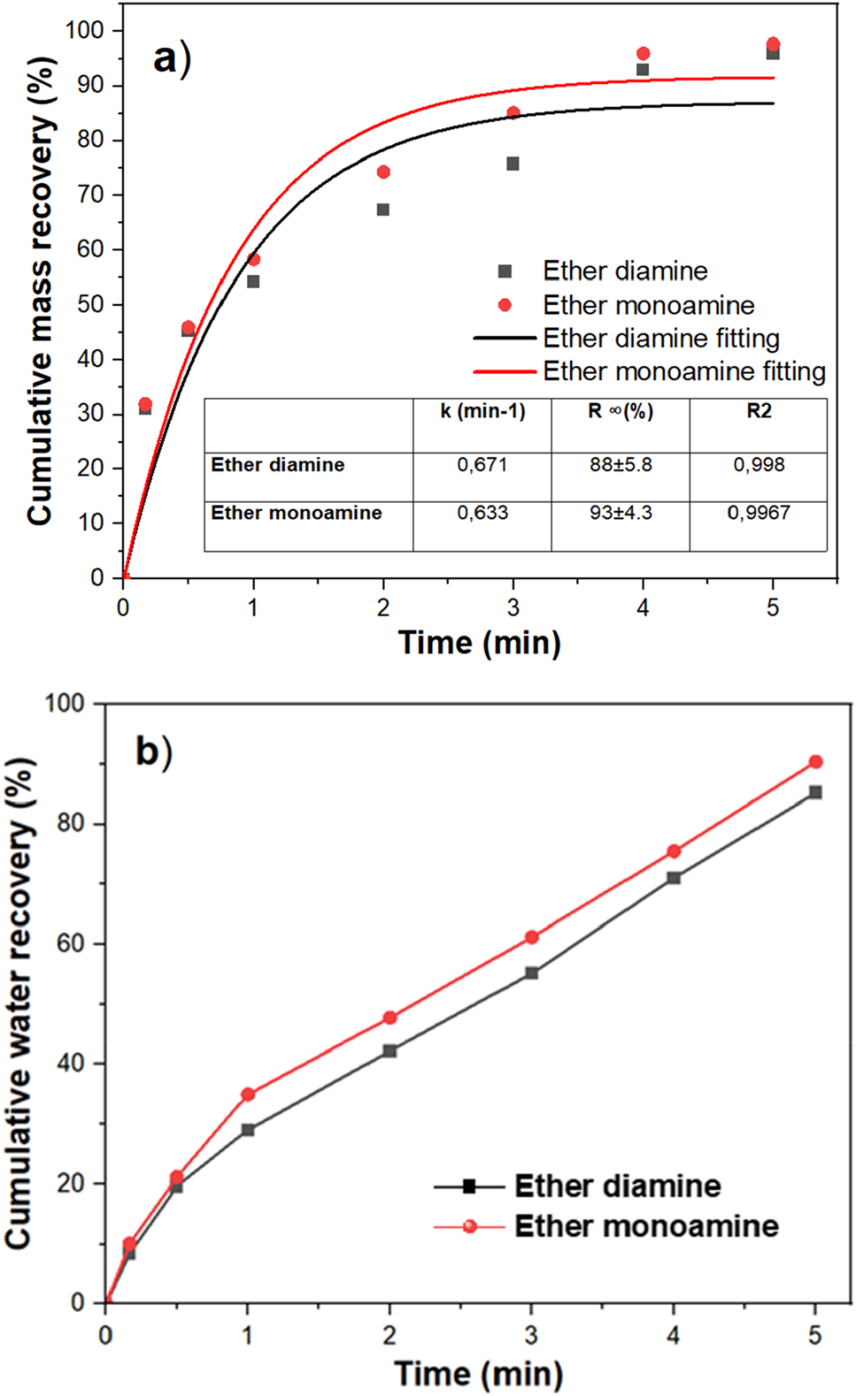
Flotation kinetics (**a**) mass recovery and (**b**) water recovery at 50 mg/L and pH 9.

The recovery-time profile depicted in Fig. 3a illustrates the flotation kinetics of quartz in the presence of ether monoamine and ether diamine. Based on the first-order model (Eq. 1), the flotation constant *k* was 0.671 min^−1^ and 0.633 min^−1^ for ether diamine and ether monoamine, respectively. Since the kinetic rate is roughly similar for both collectors and the air flow rate plus turbulence was the same during the execution of all flotation experiments, it is plausible to infer that the efficiency of particle collection by bubbles (collision, attachment, and detachment) have occurred with the same efficiency in both cases (ether monoamine versus ether diamine). On the other hand, because the flotation experiments conducted with both collectors produced a froth layer that showed different characteristics (bubbles’ geometry, volume, and water content), in Fig. 2, the higher maximum recovery (${R}_{\infty }$) shown by ether monoamine (${R}_{\infty }=$ 93 ± 4.3%) compared to ether diamine (${R}_{\infty }=$ 88 ± 5.8%) is probably due to the differences in the froth phase. The froth phase is critical in flotation performance as it dictates selectivity by retaining the hydrophobic particle whilst rejecting the hydrophilic particles. A more voluminous and wet froth produced by ether monoamine could have prevented floated particles from dropping back into the solution. This also points to the three-phase phenomena of froth flotation encompassing a gas, liquid, and even more than one solid, as described in the literature^23,24^. Furthermore, according to the results depicted in Fig. 3b, the higher water recovery shown by ether monoamine (90.4%) compared to ether diamine (85.2%) could also have contributed to the drag of very fine particles into the froth layer by entrainment. The transport of both hydrophobic and hydrophilic particles (solid phase) depends on the water recovery (liquid phase) to reach the froth phase (gas phase) either via true flotation or entrainment^25,26^.

#### Mixed mineral flotation

**Figure 4:**
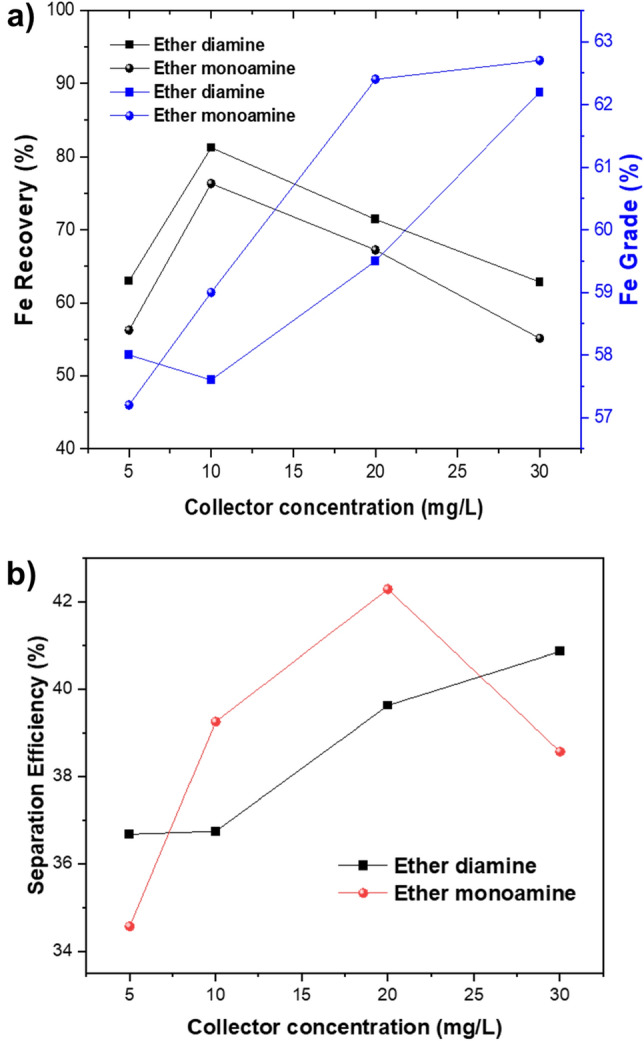
Variation of metallurgical performance as a function of collector concentration and fixed depressant at pH 9.

Mixed mineral flotation tests were carried out to assess the performance of the two ether amines on a model ore composed of magnetite (67%) plus quartz (33%), resulting in a magnetite: quartz mass ratio of 2:1). The flotation feed of the mixture had a Fe grade of 47.8% and a Si grade of 13.8%. The experimental test results shown in Fig. 4a depict the variation of Fe recovery and grade as a function of different collector concentrations at pH 9 in the presence of 100 mg/L of depressant (translating to 1000 g/t). In this particular flotation system, the magnetite concentrate is the sunken product, whereas the quartz particles are directed to the froth (tailings). Regardless of the type of collector used in the flotation experiments, the maximum Fe recovery was achieved at 10 mg/L of ether monoamine (R = 76.3%) or ether diamine (81.3%), accompanied by Fe grades that do not meet market specifications for steelmaking (pellet feed): ether diamine and ether monoamine were 57.6% for ether diamine and 59.0% for ether monoamine. For a surfactant concentration greater than 10 mg/L, the recovery of Fe in the sunken product decreased with collector concentration (from 10 to 30 mg/L), whereas Fe-content increased to values greater than 62%. Furthermore, in Fig. 4a, it is observed that diamine ether exhibits a lower ability to flounder quartz than monoamine ether since the sunken products (magnetite concentrates) produced are poorer in Fe (and richer in SiO_2_) than the concentrates produced by ether monoamine. Regarding magnetite/quartz separation selectivity (Fig. 4b), the maximum separation efficiency (SE) for ether monoamine versus ether diamine was achieved at the collector concentration of 20 mg/L (SE ~ 42.5%) versus 30 mg/L (SE ~ 41.0%), respectively. All of these results indicate that ether monoamine performs better than ether diamine to concentrate magnetite via reverse cationic flotation of quartz.

### Zeta potential

**Figure 5:**
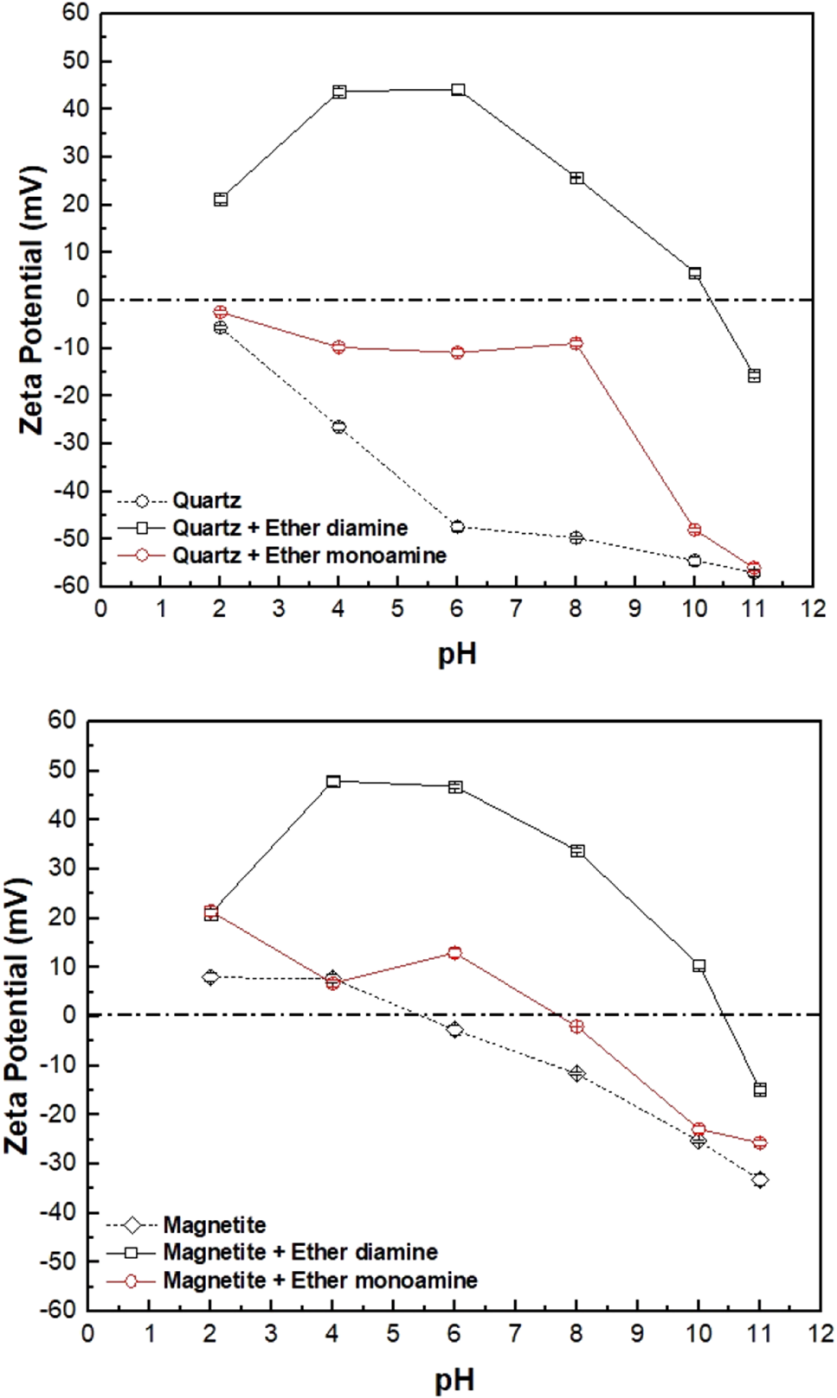
Zeta potentials of quartz and magnetite at different pH values with and without the presence of 30 mg/L of ether diamine and ether monoamine (Eh: − 0.02 to + 0.01 V).

Based on zeta potential measurements, the IEP (isoelectric point) of bare quartz and magnetite occurred at pH 2 and pH ~ 5.4, respectively (Fig. 5). These results are in good agreement with the IEP reported in the literature that ranges from 1.8 to 3.0 and 4.9 to 5.6 for natural quartz and magnetite, respectively^28–32^. Since the flotation experiments were carried out at pH 9, the zeta potential of quartz and magnetite particles in the presence of a fixed amount of collector (30 mg/L) at pH 9 deserves special attention. According to the results depicted in Fig. 5a, the quartz zeta potential, which is approximately − 50 mV for the bare particles, changes to − 24 mV in the presence of ether monoamine and to + 10 mV in the presence of ether diamine. Such a variation in the magnitude and sign of the zeta potential of quartz is evidence of collector adsorption onto the quartz/water interface. However, at a surfactant concentration of 30 mg/L and pH 9, both collectors promote quartz recovery greater than 90% (Fig. 2). From the results depicted in Fig. 5b, one can infer that the magnetite zeta potential, which is about − 20 mV for bare particles, shifts to − 10 mV in the presence of ether monoamine and to + 20 mV in the presence of ether diamine. These results indicate that collector cationic species can adsorb onto the magnetite/solution interface because they can modify the magnitude and sign of the zeta potential of magnetite particles. Therefore, using a depressant (caustic starch) is mandatory to promote selective separation between magnetite and quartz via froth flotation.

**Figure 6:**
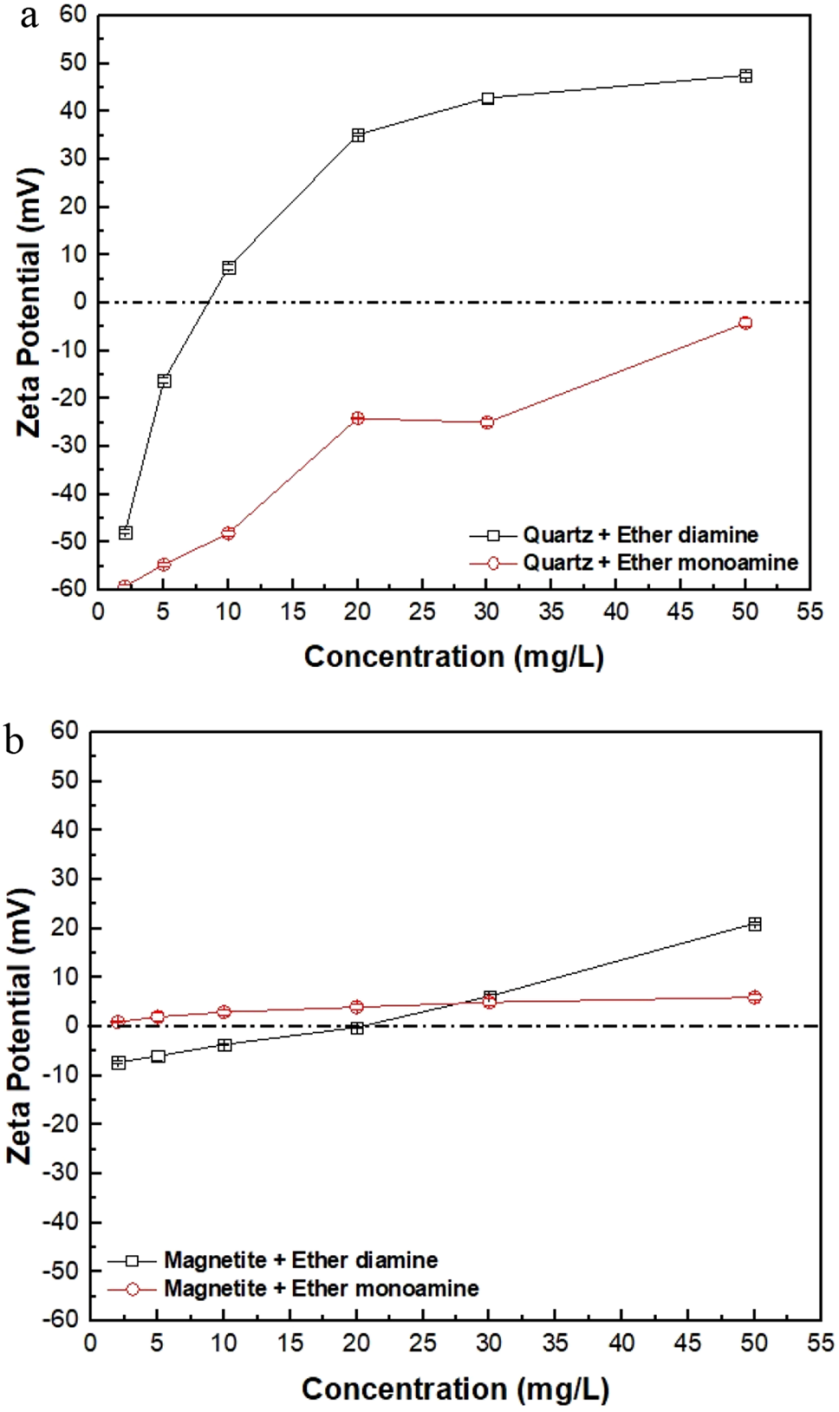
Zeta potentials of quartz and magnetite at varied concentrations of ether diamine and ether monoamine at pH 9 (Eh: − 0.02 to + 0.01 V).

Figure 6 shows the zeta potential of quartz (Fig. 6a) and magnetite (Fig. 6b) versus the increasing collector concentration (from 2.5 to 50 mg/L) at pH 9. Regarding ether monoamine, the zeta potential of the quartz particles (Fig. 6a) becomes increasingly less negative as the collector concentration increases from 2.5 to 50 mg/L, while the zeta potential of the magnetite particles (Fig. 6b) increases slightly (from ~ 0 to ~  + 5 mV) in the same collector concentration range. Considering ether diamine, the zeta potential of quartz particles (Fig. 6a) decreases from -50 mV to zero as the collector concentration increases from 2.5 to 7.5 mg/L. As collector concentration increases from 10 to 50 mg/L, zeta potential increases from approximately + 5 to + 50 mV. However, the zeta potential of magnetite particles decreases from approximately − 8 mV to zero as the collector concentration increases from 2.5 to 20 mg/L but increases to + 20 mV when the collector concentration is 50 mg/L. For both ether monoamine and ether diamine, changes in the magnitude and sign of zeta potential of magnetite and quartz versus collector concentration provide evidence of collector adsorption onto the mineral/solution interface. Changes in magnetite zeta potential versus collector concentration at pH 9 (Fig. 6b) justify using a depressant to achieve selective separation between quartz and magnetite through reverse cationic flotation.

### Turbidity

**Figure 7:**
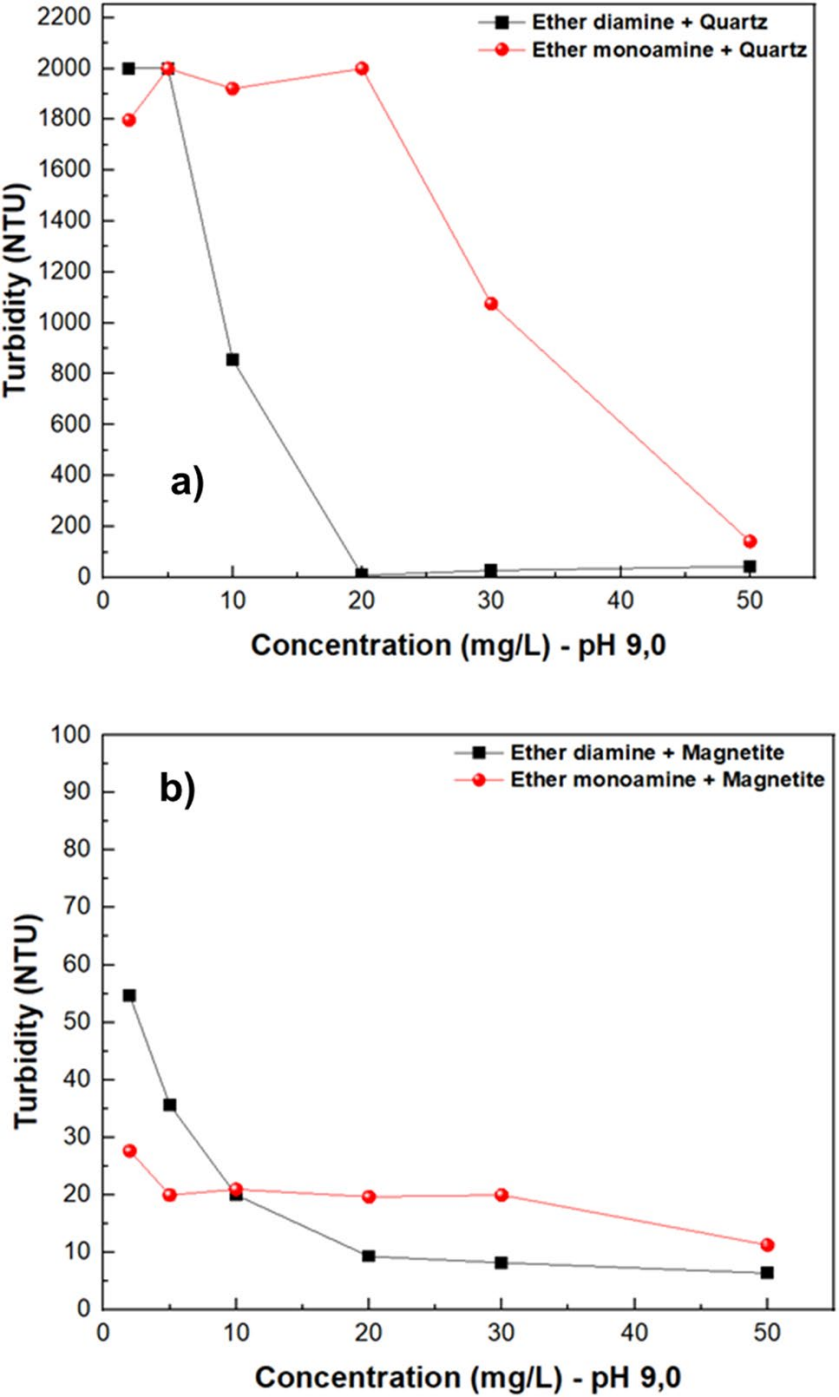
Turbidity of quartz and magnetite suspensions as a function of the concentration of ether diamine and ether monoamine at pH 9.0.

Particles can aggregate through three different mechanisms: coagulation, flocculation, and agglomeration. Electrolytes must be able to induce coagulation, which must decrease the existing electrostatic repulsion between the suspended particles. Flocculation occurs after the adsorption of polymers (flocculants) onto the mineral/solution interface, whereas agglomeration results from particle–particle interaction via dispersive forces. Because the latter can be a consequence of previous collector adsorption at the mineral/water interface, the aggregation/dispersion status of an aqueous mineral suspension can be used to assess the extension of collector adsorption onto the mineral/solution interface, mostly when particles are sufficiently small to exhibit the lowest sedimentation rates, such as the fine (− 20 $\mathrm{\mu m}$) particles used in our experimental system. In this study, a qualitative assessment of the extension of ether monoamine versus ether diamine to the surface of quartz and magnetite particles was carried out by measuring the turbidity of the supernatant of diluted mineral suspensions at pH 9 (flotation pH) in the presence of different collector concentrations (ether monoamine versus ether diamine) concentrations: 2, 5, 10, 20, 30, and 50 mg/L. The results are shown in Fig. 7. According to the results depicted in Fig. 7a, the supernatant turbidity of the quartz suspensions, previously treated with ether monoamine or ether diamine, decreased as the collector concentration increased. The same behavior was also observed for magnetite (Fig. 7b). However, the maximum value of supernatant turbidity for quartz (2000 NTU in Fig. 7a) versus magnetite (55 NTU in Fig. 7b) is possibly due to the pronounced gap of specific gravity exhibited by both mineral species: 2648 kg/m^3^ (quartz) versus 5171 kg/m^3^ (magnetite). The trend in supernatant turbidity (ST) observed for quartz (Fig. 7a) versus magnetite (Fig. 7b) indicates that ST for both mineral suspensions decreased with the addition of collectors to the system and is an indirect indication of particle–particle interaction via hydrocarbon chains of collector films adsorbed at the particle/ solution interface. The quartz supernatant showed the lowest turbidity values (ST $\le$ 200 NTU) at collector concentrations greater than 20 mg/L for ether diamine and 50 mg/L for ether monoamine. In addition, magnetite supernatant showed the lowest turbidity values (ST < 20 NTU) at collector concentrations greater than 10 mg/L for ether diamine and 50 mg/L for ether monoamine.

### Wettability

**Figure 8:**
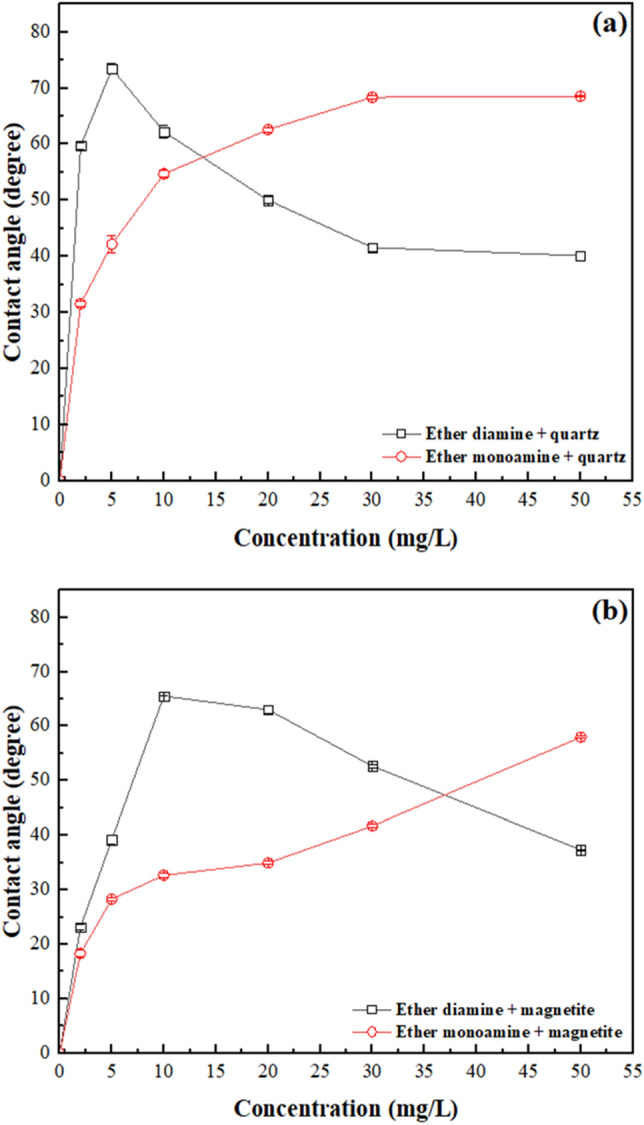
The contact angle of quartz and magnetite with ether amines with different concentrations at 22 ± 1 °C.

**Figure 9:**
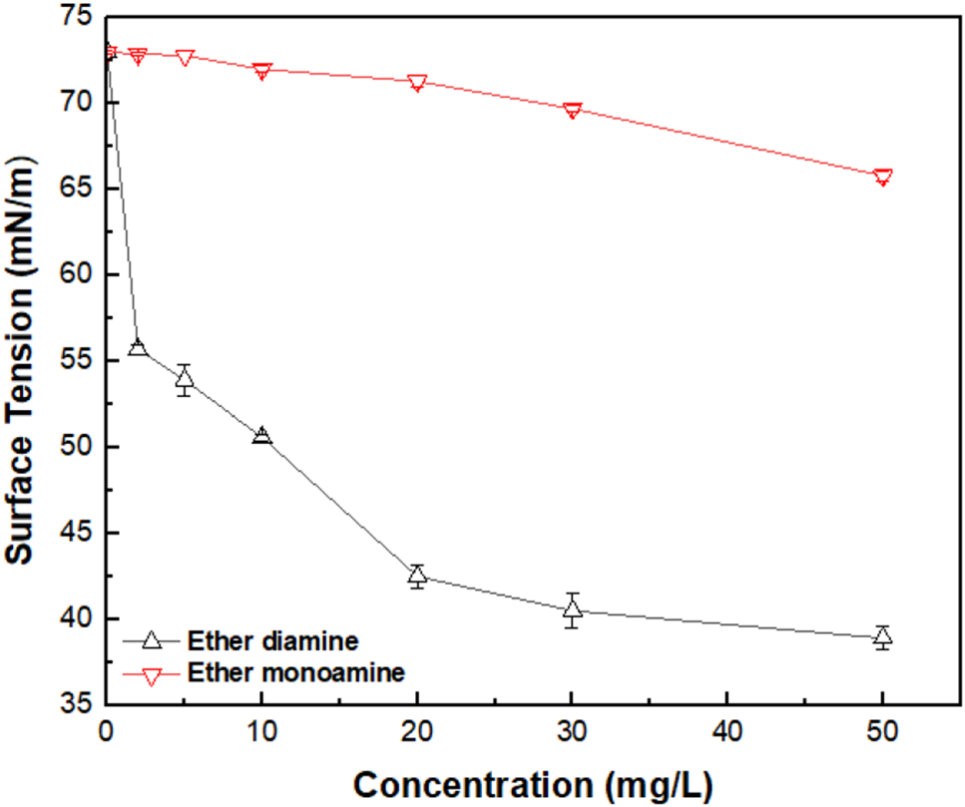
Surface tension (${\gamma }_{LG}$) of aqueous solutions versus concentration of ether monoamine and ether diamine (pH 9, 22 °C $\pm$ 1 °C).

Results of contact angle measurements (Fig. 8) and surface tension of collector solution (Fig. 9) were used to calculate the magnitude of the work of adhesion (${W}_{a}$) by using Eq. (7), the work of cohesion (${W}_{c}=2{\gamma }_{LG})$, as well as the free energy of particle/bubble attachment (${\Delta G}_{attachment}$) via Eq. (8). To start comparing the influence of both collectors on the wettability of quartz and hematite, one must account for the results displayed in Fig. 9: as collector concentration increased from 0 to 50 mg/L, ${\gamma }_{L/G}$ of the ether monoamine aqueous solution decreased slightly (from 73.0 to 65.8 mN/m) compared to ${\gamma }_{L/G}$ of the diamine aqueous solution (from 73.0 to 38.9 mN/m). The greatest ${\gamma }_{L/G}$ reduction observed for ether diamine compared to ether monoamine indicates that the former was much more surface active than the latter, and this striking feature cast the wettability of quartz and magnetite in the presence of those collectors.

Results of contact angle ($\theta$) measurements by captive bubble method for quartz and magnetite (Fig. 8) showed that in the presence of ether monoamine, strictly increasing values of $\theta$ for quartz (from 13° to 70°) and magnetite (from 12° to 54°) were observed as collector concentration augmented from 0 to 50 mg/L. Considering ether diamine within the same range of collector concentration (0–50 mg/L), the magnitude of $\theta$ showed a maximum value for quartz ($\theta$ = 74°) and magnetite ($\theta$ = 66°) at collector concentrations of 5 and 10 mg/L, respectively. This way, as the diamine concentration became higher than 5 mg/L for quartz and higher than 10 mg/L for magnetite, $\theta$ decreased steadily for either quartz (from 74° to 40°) or magnetite (from 66° to 37°), although the magnitude of $\theta$ was sufficiently high to promote the flotation of ultrafine particles (${d}_{50}$=7.5 $\mathrm{\mu m}$)^33^. These results corroborated the high floatability of quartz depicted in Fig. 2 and also supported the need for corn starch to depress magnetite, increasing the separation efficiency (Fig. 4). Considering that an excess of ether diamine in solution was able to decrease the hydrophobicity ($\theta$) of quartz (concentration > 5 mg/L) and magnetite (concentration > 10 mg/L), practitioners should exert a stricter control of collector dosage in industrial flotation circuits when ether diamine was adopted as a collector.

**Figure 10:**
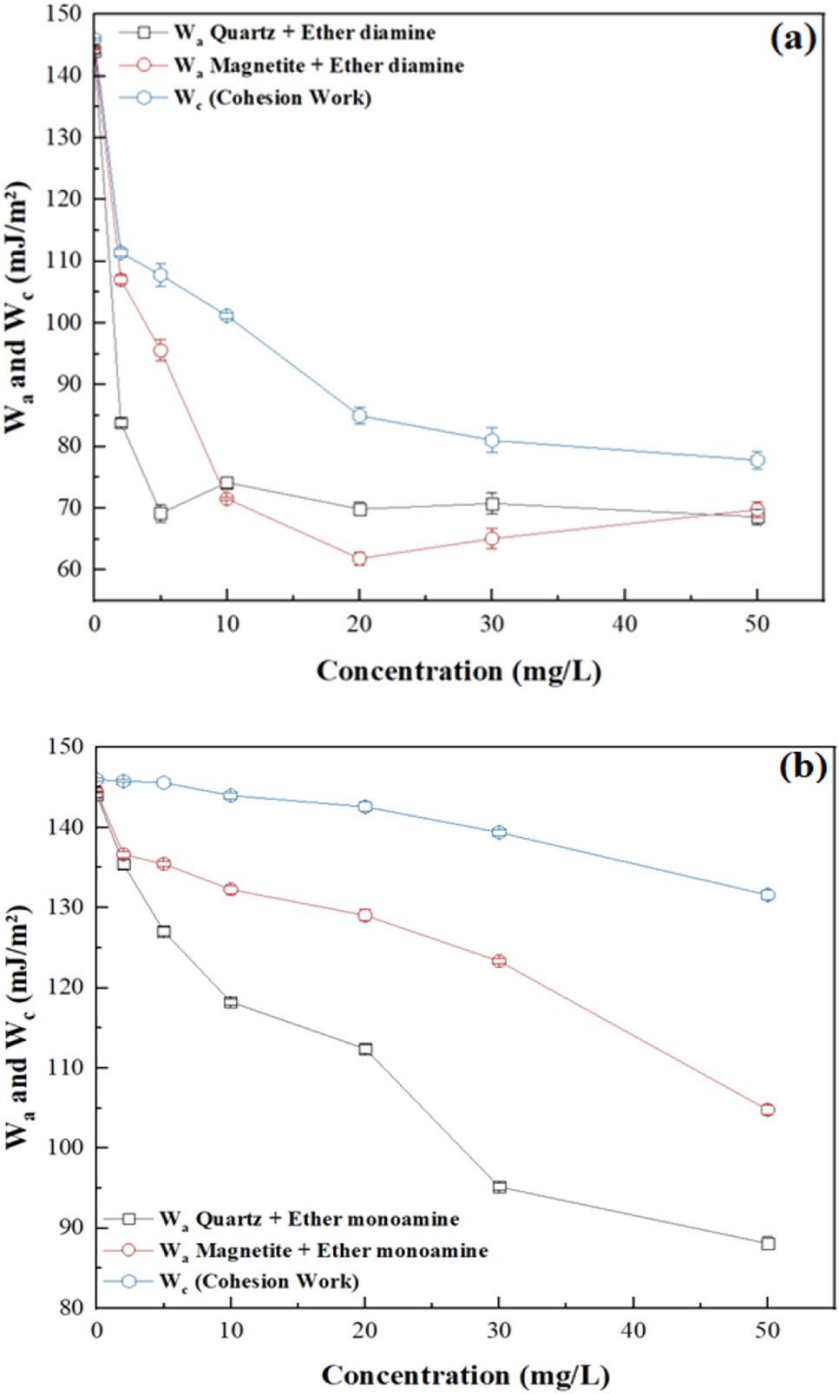
Work of adhesion of water to quartz and magnetite plus work of cohesion of the aqueous solution in the presence of (**a**) Ether diamine and (**b**) Ether monoamine (pH 9, temperature = 22 °C $\pm$ 1 °C).

The tendency of water to be displaced from a mineral surface by an air bubble was controlled by the magnitude of the work of adhesion ($${W}_{a}$$) versus the work of cohesion ($${W}_{c}$$): the naturally hydrophilic character of quartz and magnetite was preserved when $${W}_{a}$$ > $${W}_{c}$$, whereas the hydrophobic character was achieved whenever $${W}_{a}$$ < $${W}_{c}$$, after collector adsorption onto the mineral/solution interface. This way, according to values displayed in Table 3, at room temperature, bare particles of quartz ($${W}_{a}$$ = 470 mJ/m^2^) were fully wetted by water ($${W}_{c}$$ = 146 mJ/m^2^), since $${W}_{a}$$ >$${ W}_{c}$$. On the other hand, to float quartz (and magnetite), it was necessary to add a collector to the system aiming to meet the condition $${W}_{a}$$ <$$ { W}_{c}$$, which was verified for either ether diamine (Fig. 10a) or ether monoamine (Fig. 10b) whenever collector concentration (c) was higher than zero. However, the performance of monoamine versus diamine was very distinct: while $${W}_{a}$$ for quartz/solution and magnetite/solution decayed smoothly with the concentration of ether monoamine, it showed a steep decay with the concentration (c) of ether diamine, showing a minimum value for quartz ($${W}_{a}$$ = 69 mJ/m^2^) at c = 5 mg/L and for magnetite ($${W}_{a}$$ = 72 mJ/m^2^) at c = 10 mg/L. For higher concentrations of ether diamine, the magnitude of $${W}_{a}$$ for both minerals tended to stabilize in a plateau of 70 mJ/m^2^. These results also indicated that, unlike ether monoamine, the ability of ether diamine to promote the hydrophobic character was more pronounced at 5 mg/L for quartz and 10 mg/L for magnetite.

**Table 3:** Surface tension of aqueous solutions of ether monoamine and diamine at (22 $\pm 1$) °C.

Concentration (mg/L)	Ether diamine (mN/m)	Ether monoamine (mN/m)
2	55.7 ± 0.3	72.9 ± 0.1
5	53.9 ± 0.9	72.8 ± 0.0
10	50.6 ± 0.2	72.0 ± 0.2
20	42.5 ± 0.7	71.3 ± 0.3
30	40.5 ± 1.0	69.7 ± 0.2
50	38.9 ± 0.7	65.8 ± 0.3

**Figure 11:**
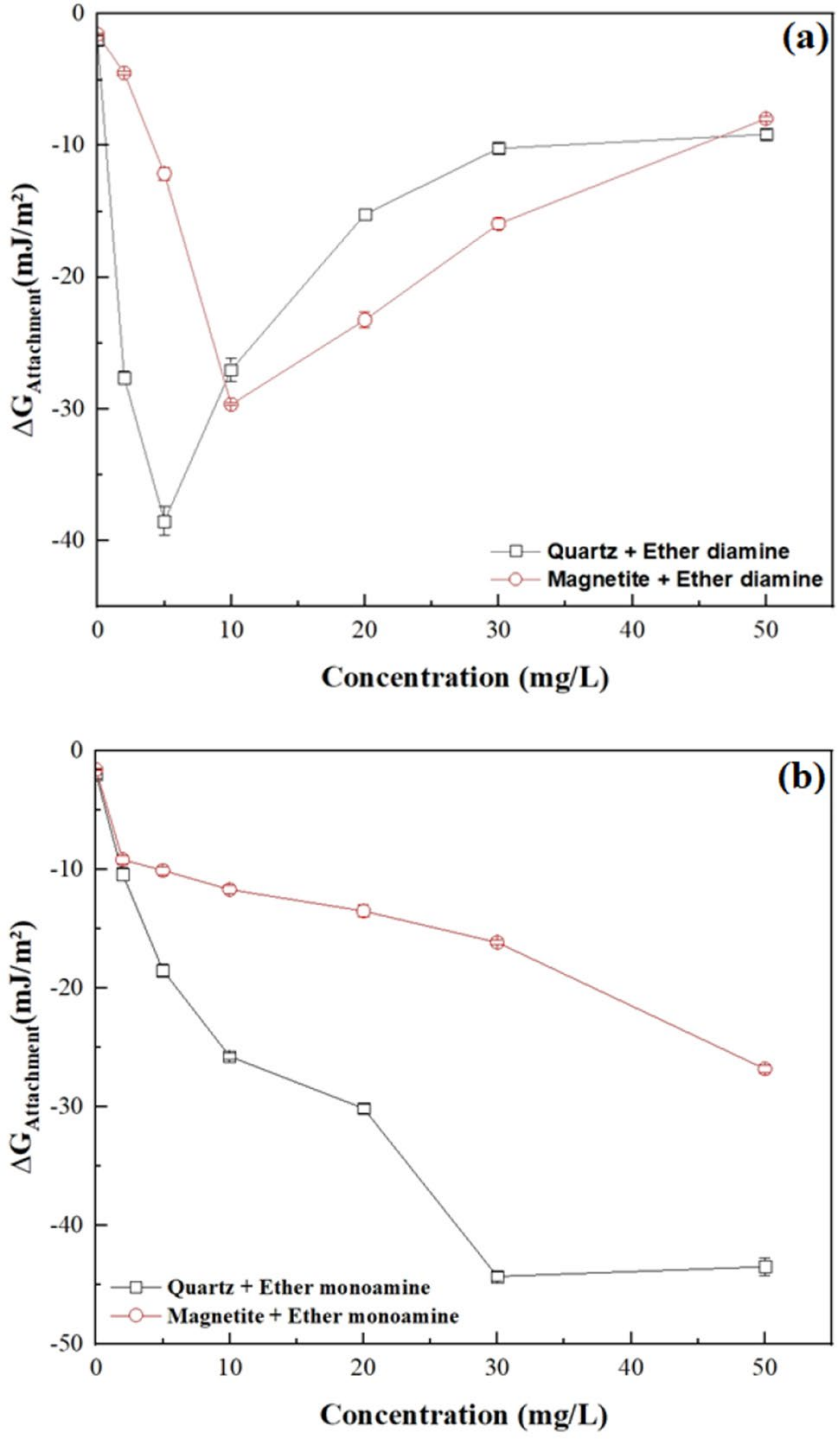
Free energy of particle-bubble attachment for quartz and magnetite in the presence of (**a**) Ether diamine and (**b**) Ether monoamine (pH 9, temperature = 22 °C $\pm$ 1 °C).

This trend was also shown in Fig. 11, where the minimum (most negative) value of the free energy of particle/bubble attachment for quartz (${\Delta G}_{attachment}$ = − 38.5 mJ/m^2^) and magnetite (${\Delta G}_{attachment}$ = − 29.7 mJ/m^2^) occurred at the same ether diamine concentration, which promoted the highest values of contact angle for either quartz ($\theta$ = 74° at 5 mg/L) or magnetite ($\theta$ = 66° at 10 mg/L). Conversely, for the less surface-active collector (monoamine), the magnitude of ${\Delta G}_{attachment}$ was strictly decreasing for both minerals as collector concentration increased from 0 to 50 mg/L.

### FTIR spectra analysis

**Figure 12:**
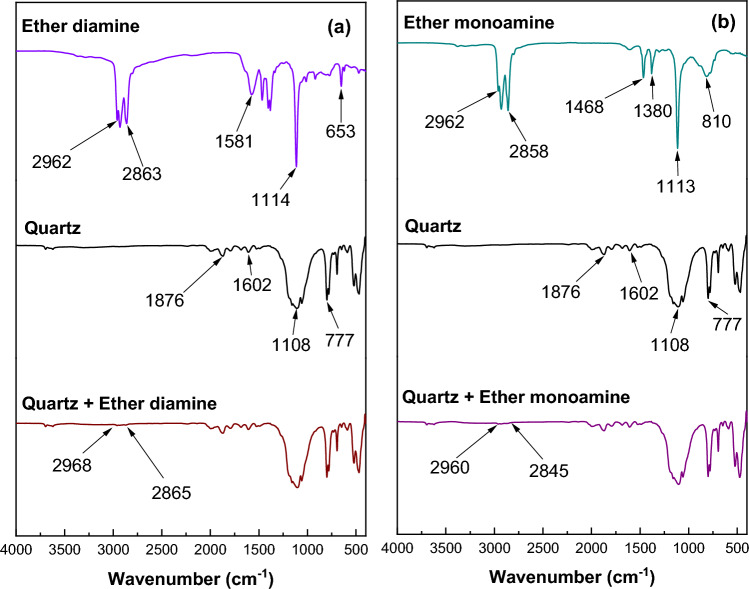
FTIR spectra for (**a**) ether diamine and (**b**) ether monoamine before and after treatment.

FTIR analyses illustrated (Fig. 12) that for diamine ether, characteristic peaks emerged at 2962 cm^−1^ and 2863 cm^−1^, which were attributed to the CH_2_ stretching bond of acyclic compounds^29^. The peak at 1581, 1114, and 653 cm^−1^ could be attributed to the bending of NH_2_ or NH bonds^29–31^. Similar characteristic peaks were observed in ether monoamine at 2962 cm^−1^ and 2858 cm^−1^, which were attributed to the CH_2_ stretching bond of acyclic compounds^35^. The peak at 1468, 1113, and 810 cm^−1^ could be attributed to the bending of NH_2_ or NH bonds^35–37^. When comparing the two amines, they vary on the 1600–600 cm^−1^ peaks. After treatment with both amines, a characteristic peak was observed on the quartz surface. After treating the quartz with ether diamine, the characteristic peak of OH at 2962 cm^−1^ shifted to 2865 cm^−1^, as observed in the treated quartz. Similarly, the characteristic peak of OH at 2962 cm^−1^ shifted to 2960 cm^−1^ for ether monoamine, as observed on the treated quartz. Both findings are consistent with the findings of Liu et al.^35^. Both amines could be adsorbed more on the quartz surface and agree with the finding documented by Huang et al. and Liu et al.^35,36^ about the amine adsorption on silicates.

## Conclusions

Exploring the flotation of ultrafine magnetite from quartz (− 20 µm) by ether diamine 'Lilaflot® 811 M' and ether monoamine 'Lilaflot® 919' indicated that both biodegradable collectors could efficiently separate these two minerals by reverse cationic flotation of quartz. Single mineral flotation indicated that at pH 9 both collectors promoted high quartz recovery (95.9% ether diamine versus 97.7% ether monoamine), and relatively similar flotation kinetics (*k* was 0.671 min^−1^ and 0.633 min^−1^ for ether diamine and ether monoamine, respectively). Turbidity analyses demonstrated that quartz particles tend to aggregate above 10 mg/L of collector concentration. However, under that condition, the magnetite particles aggregated much faster than the quartz, which improved the magnetite depression and separation process. Ether monoamine indicated a higher water recovery (90.4 vs. 85.2%) and was accompanied by a generally higher separation efficiency. Various surface analyses revealed that collectors are mostly adsorbed on the quartz surface, whereas a weak physical interaction was dominant for magnetite. An increase in the concentration of diamine ether was shown to decrease its contact angle on the quartz surface (from 40.1° to 37.3°). Moreover, surface tension assessments indicated that the surface energy decreased more strongly in the presence of ether diamine vs. ether monoamine, which made it a more selective collector (higher grade and lower recovery: lower separation efficiency).

## Data Availability

All data generated or analyzed during this study are included in this published article.
